# A Policy Impact Case Study Using Real World Data from Welsh Government Fuel Poverty Schemes to Inform Scheme Design.

**DOI:** 10.23889/ijpds.v7i3.1894

**Published:** 2022-08-25

**Authors:** Sarah Lowe, Sian Morrison-Rees

**Affiliations:** 1 Administrative Data Research Unit – Wales, Welsh Government; 2 ADR Wales, Swansea University

## Objectives

To reduce fuel poverty in Wales: the Welsh Government developed schemes to provide energy efficiency improvements to lower income households.

To inform scheme design: investigate health impacts by linking scheme data to health records.

Presented objective: to demonstrate how research findings using real world data can impact policy focus.

## Approach

The research was conducted by an independent researcher at Swansea University who co-produced research questions with the Welsh Government Fuel Poverty Policy Team.

A longitudinal dataset was created by linking anonymised ‘Warm Homes: Nest’ improvements data to residents’ routine health records in the SAIL Databank at Swansea University. We examined recipient health before and after intervention compared with controls.

A high-level policy briefing and research report were published in the Welsh Government Social Research – Analysis for Policy series.

Findings were used to design and pilot new eligibility criteria to capture low-income individuals with a respiratory, circulatory or mental health condition.

## Results

This presentation will describe the policy impact pathway from initial discussions with policymakers to real world change, including:

- securing ESRC funding for a Knowledge Transfer Fellowship, which included a 2013 data linking demonstration project…
- …which allowed funding to be secured for a 2015-18 research project focused on the impact of improvements on recipient health…
- …which published emerging findings in 2016…
- …and substantive findings in 2017, showing a significant positive impact of improvements on recipient health…
- …which policymakers used to design a pilot to test ways to widen eligibility criteria to include individuals on a low income with a respiratory, circulatory or mental health condition…
- …which led to scheme criteria being widened in 2019.

By 2021, 25% of recipients entered the scheme via the ‘health route’.

## Conclusion and Relevance

By delivering research findings generated using linked real world data, and focused on questions co-produced with policymakers, researchers can successfully impact the design and implementation of government policy, thereby improving the lives of people in the real world - in this case, the health of the citizens of Wales.

